# Regulator of G protein signaling 4 inhibits human melanoma cells proliferation and invasion through the PI3K/AKT signaling pathway

**DOI:** 10.18632/oncotarget.20825

**Published:** 2017-09-11

**Authors:** Xiaotong Xue, Lihua Wang, Xianguang Meng, Jing Jiao, Ningning Dang

**Affiliations:** ^1^ School of Medicine, Shandong University, Jinan, Shandong Province, China; ^2^ Department of Dermatology, Jinan Central Hospital Affiliated to Shandong University, Jinan, Shandong Province, China; ^3^ Department of Dermatology, The Chinese People’s Liberation Army 88 Hospital, Taian, Shandong Province, China; ^4^ College of Life Science, Shandong Normal University, Jinan, Shandong Province, China

**Keywords:** RGS4, melanoma, proliferation, invasion, PI3K/Akt

## Abstract

Melanoma is a tumor produced by skin melanocytes, which has a high metastatic rate and poor prognosis. So far, plenty of work has been done on melanoma, but mechanisms underlying melanoma development have not been fully elucidated. Here we identified regulator of G protein signaling 4(RGS4) as novel therapeutic target for malignant melanoma and its regulating effect on melanoma. We found that endogenous RGS4 expression was much lower in melanoma tissues and cells. In A375 cell line with low endogenous RGS4 expression, the function of RGS4 was detected by up-regulation its expression with pcDNA3.1-RGS4 and knockdown its expression with siRNA. Our results showed that RGS4 could significantly reduce the proliferation, migration and invasion of melanoma cells. RGS4 is an important regulator for the apoptosis of melanocyte, and the apoptosis rate is significantly decreased in low RGS4 enviroment. RGS4 induced non-activation of PI3K/AKT pathway, resulting in decreased expression of E2F1 and Cyclin D1, thus constraining cell proliferation and invasion. These results were further confirmed in M14 cell lines. Collectively, our findings show that RGS4 plays an important role in multiple cellular functions of melanoma development and is valuable to be a therapeutic target.

## INTRODUCTION

Melanoma is a highly malignant tumor derived from cutaneous melanocytes. It has high metastasis rate and poor prognosis. Among them, high aggression, drug-resistance, early blood and lymphatic metastasis are the primary causes of melanoma death [1]. In the early stage of melanoma, it could be cured by resection, while in the late stage it is difficult to be treated by current therapies [2]. Once melanoma has invaded the regional lymph nodes, the 5-year survival rate cannot exceed 10% [3]. Although great progress has been made in the pathogenesis melanoma and related signaling pathways, the precise mechanism of malignant melanoma development has not yet been entirely explained .To improve the treatment of melanoma patients, more work has been done to clarify the molecular and cellular mechanisms of this disease. To date, studies have shown that the occurrence and development of melanoma are related to certain signaling pathways, such as phosphoinositide-3 kinase-serine/threonine protein kinase (PI3K/AKT), WNT/β-catenin and Ras/Raf/MEK/ERK [4–6].

The G-protein–coupled receptors (GPCRs) are the largest cell surface proteins family in eukaryotic organisms and have most diverse group of membrane receptors [7]. Signal transduction via GPCRs is the basis of most physiological processes, including vision, smell and taste, except for neurological, cardiovascular, endocrine, and reproductive functions [8, 9]. This major involvement has made the GPCR superfamily a major target for therapeutic intervention. It is estimated that a third to half of marketing drugs act by binding to GPCRs. On the contrary, GPCRs activation or expression abnormalities are intimately associated to cancer cell proliferation, tumor growth and metastasis [10].

The Regulator of G-protein Signaling (RGS) proteins, with different molecular size and multiple functions, belong to a large family that mainly acting on Gαi and Gαq subfamily GPCR-mediated signaling pathways as well as the adjustment of GPCR-mediated reaction in cells [11–13]. RGSs were initially characterized as inhibitors of signal transduction cascades induced by GPCRs due to their ability to increase the intrinsic GTPase activity of heterotrimeric G proteins [14]. As more and more evidences have illustrated the important roles of RGS proteins in physiology of multiple organ systems, they are considered as potential drug targets in diverse pathologies including CNS diseases, cardiovascular disease and diabetes [15–21]. Recent studies have revealed that many RGSs were involved in tumor development. For example, overexpression of RGS1 suppresses CXCL12-mediated migration in human plasmacytoma cells [22], epigenetic repression of RGS2 is related with the development of prostate cancer [23], overexpression of RGS5 plays an inhibitory role in human lung cancer cells through induction of apoptosis [24] and overexpression of RGS17 increased growth rate through the cyclic AMP-PKA-CREB pathway in lung tumor cells [25]. Therefore, it is suggested that RGS proteins are potential candidates for tumor diagnosis and treatment.

The regulator of G protein signaling 4 (RGS4) is a member of the B/R4 group [26]. It works as a multifunctional scaffold protein for assembling proteins involved in regulatory signal transduction. Overexpression of RGS4 protein blocks Gαi/o-mediated pathway and GPCR-induced Ca2+ signaling in the pancreatic acinar cells in mice [27]. RGS4 may inhibit the migration and invasion of breast cancer cells via down-regulating the metastasis-associated Gi-coupled receptors (PAR1 and CXCR4) signal transduction [28]. RGS4 is also found in NSCLC cells and its overexpression decreases invasion and migration by inhibiting MMP2/9 and reversing EMT [29]. These studies together demonstrate the importance of RGS4 in multiple cell functions. To analyze the development mechanism of melanoma, RGS4 is valuable to be our candidate.

## RESULTS

### RGS4 expression is remarkably decreased in melanoma tissues and cells

**Figure 1:**
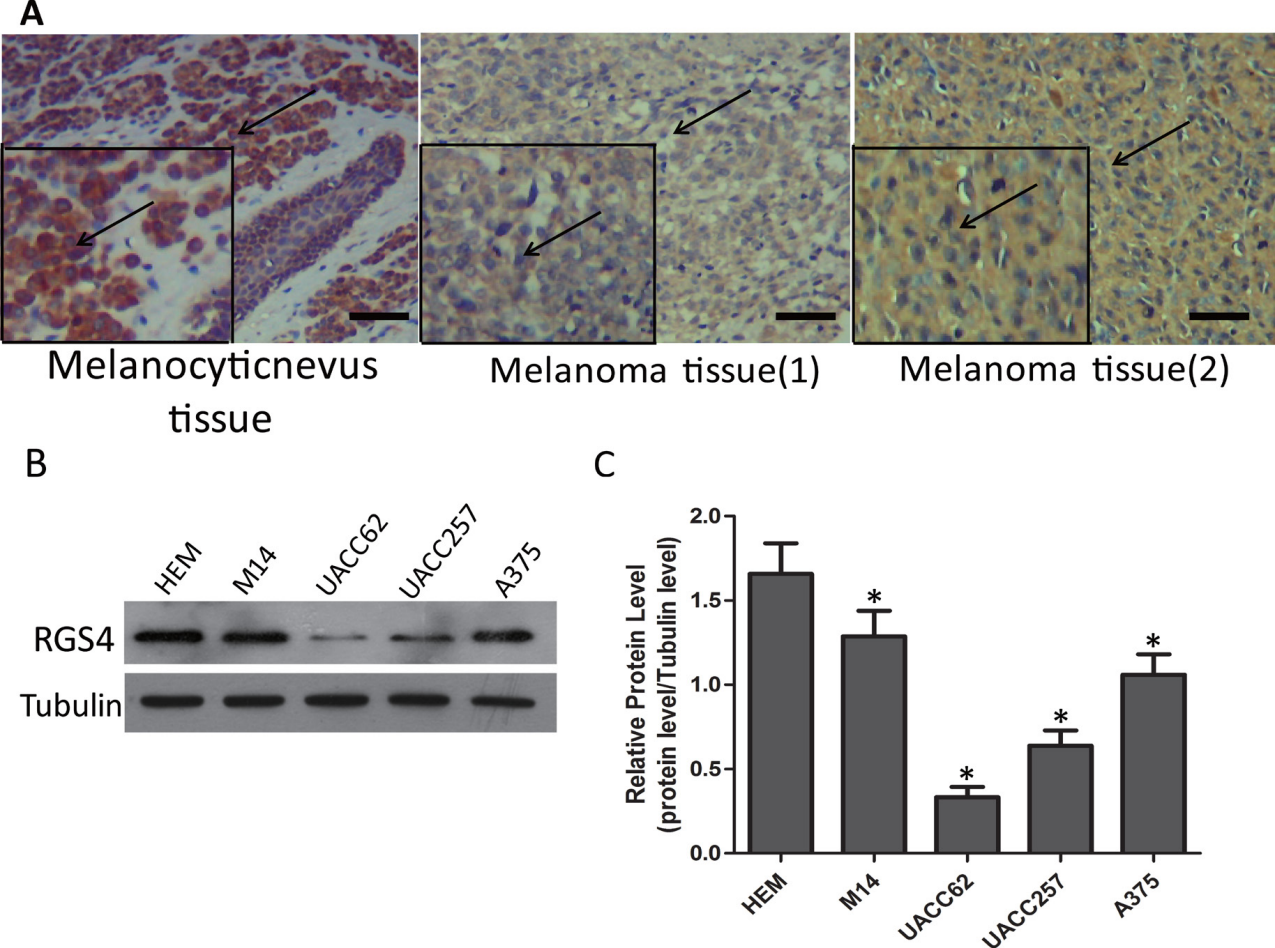
RGS4 expression is remarkably decreased in melanoma tissues and cells (**A**) Immunohistochemistry staining of RGS4 in melanocytic nevus tissues and melonoma tissues with anti-RGS4 antibody . Scale bars: 100 μm. (**B**) Western blot to show the protein levels of RGS4 varies in different normal skin cell lines and melanoma cell lines. (**C**) Quantification assay of the RGS4 bands intensity. Error bars indicate ± SD. *p < 0.05; **p < 0.01 by Student’s *t*-test.

Tissue microarray of 48 cases of sample, containing 8 cases of melanocytic nevus tissue and 40 cases of melanoma tissue, was immunohistochemically stained for RGS4 protein. There were strong immunohistochemistry staining of RGS4 in melanocytic nevus tissue while it significantly decreased in melanoma tissue (Figure 1A). As shown in Table 1, RGS4-high expression rate (75%,6/8) in melanocytic nevus tissues was obviously higher than the rate (27.5%,11/40) in melanoma tissues (*P* < 0.05). Meanwhile, by analyzing the associations with clinicopathological characteristics of melanoma, we found that RGS4 expression was significantly correlated with TNM stage (*P* < 0.01) (Table 2). To further investigate the biological functions of RGS4 in melanoma development ,we examined the expression of RGS4 in melanoma cell lines (M14, A375, UACC62, UACC257) and in normal skin cell line (Human Epidermal Melanocytes, HEM) as control by using Western blot. Our results showed that RGS4 expression level was much lower in melanoma cells than in HEM cells (Figure 1B and 1C).

**Table 1:** The expression of RGS4 in melanocytic nevus and melanoma samples

Samples	Case (*n*)	RGS4 expression		*P* value
		No or low (*n* =)	High (*n* =)	
melanocytic nevus	8	2	6	0.0103*
melanoma	40	29	11	

**Table 2:** Association of RGS4 expression with the clinicopathological characteristics of melanoma

Characteristics	Case (*n*)	RGS4 expression		*P* value
		High (*n* =)	Low or no (*n* =)	
Age				
≤ 60	27	9	18	0.2337
> 60	13	2	11	
Gender				
Male	30	9	21	0.5396
Female	10	2	8	
Tumor invasion(AJCC)				
T1–T3	9	1	8	0.1938
T4	31	10	20	
Lymphatic metastasis				
Absent	32	7	25	0.1111
Present	8	4	4	
TNM stage				
I–II	30	5	25	0.0079**
III–IV	10	6	4	

*Statistically significant difference; AJCC, American Joint Committee on Cancer.

### Overexpression of RGS4 significantly inhibits melanoma cell proliferation

**Figure 2:**
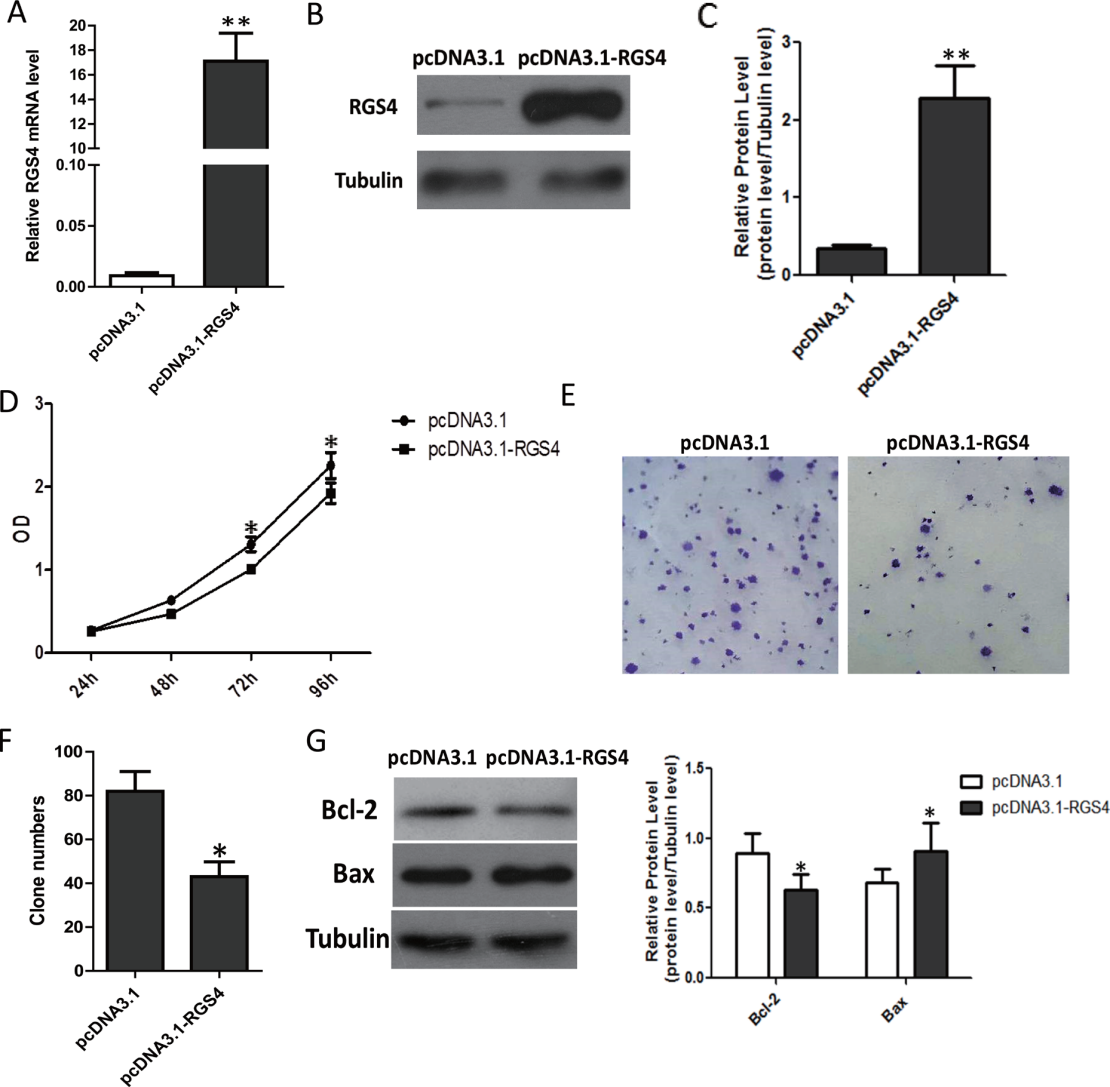
Overexpression of RGS4 significantly inhibits melanoma cell proliferation (**A**) Quantitative qRT-PCR result to show RGS4 mRNA significantly increased by pcDNA3.1-RGS4. (**B**) Western blot of RGS4 expression. (**C**) Quantification assay of the RGS4 bands intensity. (**D**) Time course curve of CCK8 assay results to show the viability change of A375 cell. (**E**) Colony-forming cells evaluated by colony formation assay. (**F**) Bar charts of positive colonies. (**G**) The expression of apoptotic-related proteins, Bcl-2, Bax, were detected in A375 cell by Western blot. Error bars indicate ± SD. *p < 0.05; **p < 0.01 by Student’s *t*-test. All the results were repeated thrice.

To investigate the biological functions of RGS4 in melanoma development, we selected A375 cell, with endogenous low RGS4 expression, to be transfected with pcDNA3.1-RGS4. The pcDNA3.1-RGS4 vector increased expression of RGS4 efficiently (Figure 2A–2C). CCK8 assay and colony formation assay evaluated the effect of the melanoma cell proliferation. CCK8 assays revealed that the growth rate was significantly decreased A375 cell as compared to control cells transfected with empty vector (pcDNA3.1) on days 3 and 4 after transfection (Figure 2D). Colony formation assays also confirmed the ability of RGS4 to suppress A375 cell proliferation (Figure 2E and 2F). In addition, the expression of anti-apoptotic protein Bcl-2 decreased while the apoptotic protein Bax increased in pcDNA3.1-RGS4 transfected cells compared with the positive control, further demonstrating that overexpression of RGS4 can inhibit A375 cell proliferation (Figure 2G).

### Overexpression of RGS4 inhibits melanoma cell migration and invasion

**Figure 3:**
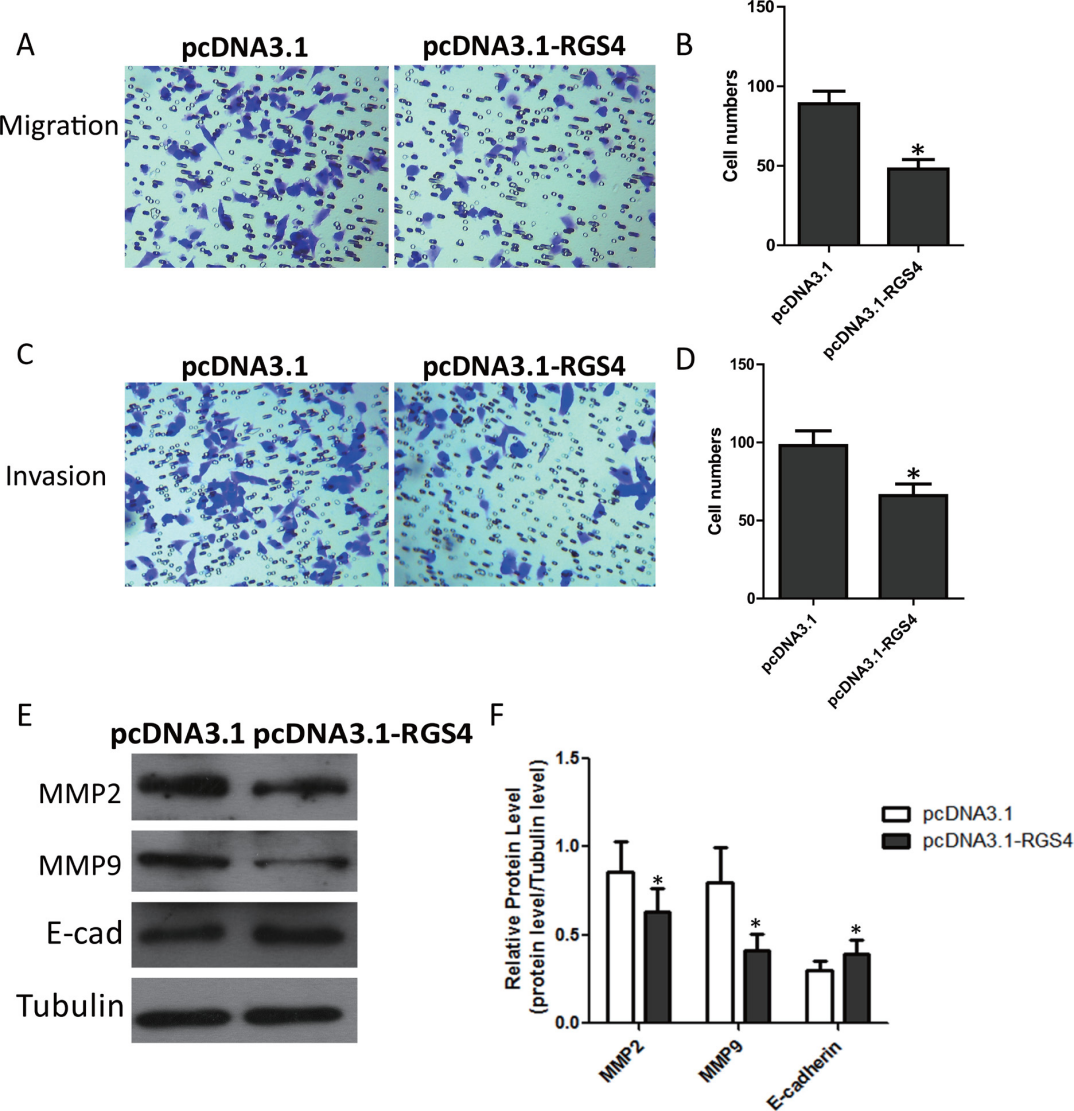
Overexpression of RGS4 inhibits melanoma cell migration and invasion (**A** to **D**) Trans-well assays ± Matrigel were performed using A375 cell transfected with pcDNA3.1-RGS4 and corresponding pcDNA3.1 vector, respectively. (**E** and **F**) The effect of RGS4 up-regulation on the expression of MMP2, MMP9 and E-cadherin was examined by Western blot. Error bars indicate ± SD. *p < 0.05; **p < 0.01 by Student’s *t*-test. All the results were repeated thrice.

Trans-well assays were performed to investigate RGS4 effect on the migration and invasion of cancer cells *in vitro*. Trans-well assays ± Matrigel demonstrated that A375 cell transfected with pcDNA3.1-RGS4 vector had lower invasive activity than control cells (Figure 3A–3D). To elucidate the molecular mechanism of the inhibition of melanoma cell invasion and migration under high level of RGS4, we detected the effect of RGS4 on the expression of matrix metalloproteinases (MMPs), the crucial molecules in cell invasion. Western blot results showed that the protein levels of MMP2 and MMP9 were notably down-regulated with overexpression of RGS4. In addition, as epitheliale mesenchymal transition (EMT) has the key role in cell invasion and migration, we further detected the effect of RGS4 on the expression of EMT markers E-cadherin. As expected, up-regulation of E-cadherin was observed in RGS4 overexpression A375 cell, demonstrating that overexpression of RGS4 inhibits melanoma cell migration and invasion (Figure 3E and 3F).

### Reduction of RGS4 significantly promotes melanoma cell proliferation

**Figure 4:**
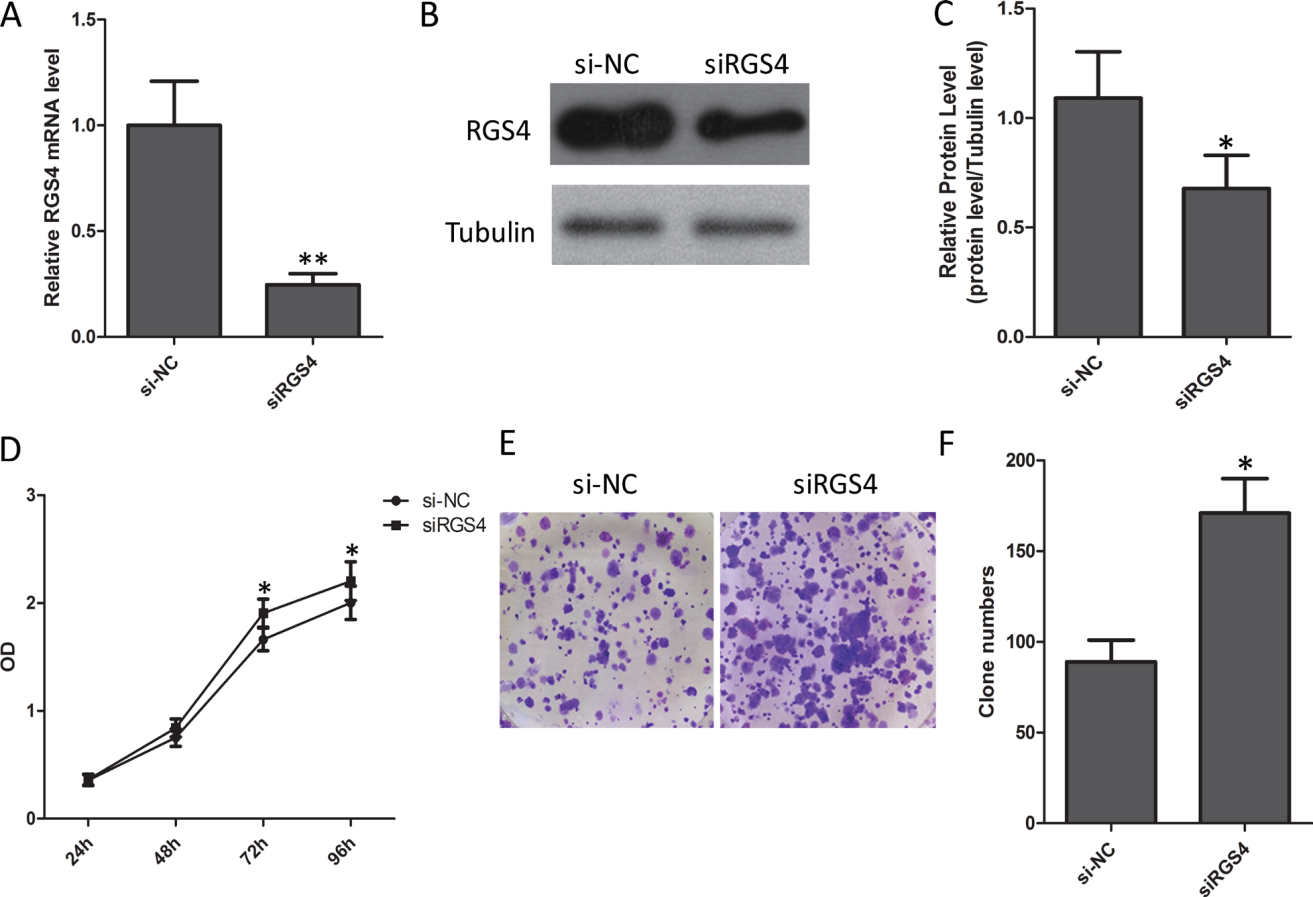
Reduction of RGS4 significantly promotes melanoma cell proliferation (**A**) Quantitative qRT-PCR result to show RGS4 mRNA significantly decreased by siRNA. (**B**) Western blot of RGS4 expression. (**C**) Quantification assay of the RGS4 bands intensity. (**D**) Time course curve of CCK8 assay results to show the viability change of A375 cell. (**E**) Colony-forming cells evaluated by colony formation assay. (**F**) Bar charts of positive colonies.Error bars indicate ± SD. *p < 0.05; **p < 0.01 by Student’s *t*-test. All the results were repeated thrice.

A375 cell with low endogenous RGS4 expression was selected for the transfer of siRGS4. The siRGS4 decreased expression of RGS4 efficiently (Figure 4A–4C). CCK8 and colony formation assay were performed to evaluate whether the reduction of RGS4 may affect the proliferation of melanoma cell. As CCK8 assay shows, low level of RGS4 significantly promoted A375 cell viability (Figure 4D). At the same time, the analysis of colony formation showed that the transfection of siRGS4 caused higher rate proliferation of A375 cell than si-NC transfection control (Figure 4E and 4F).

### Low expression of RGS4 increases melanoma cell migration and invasion

**Figure 5:**
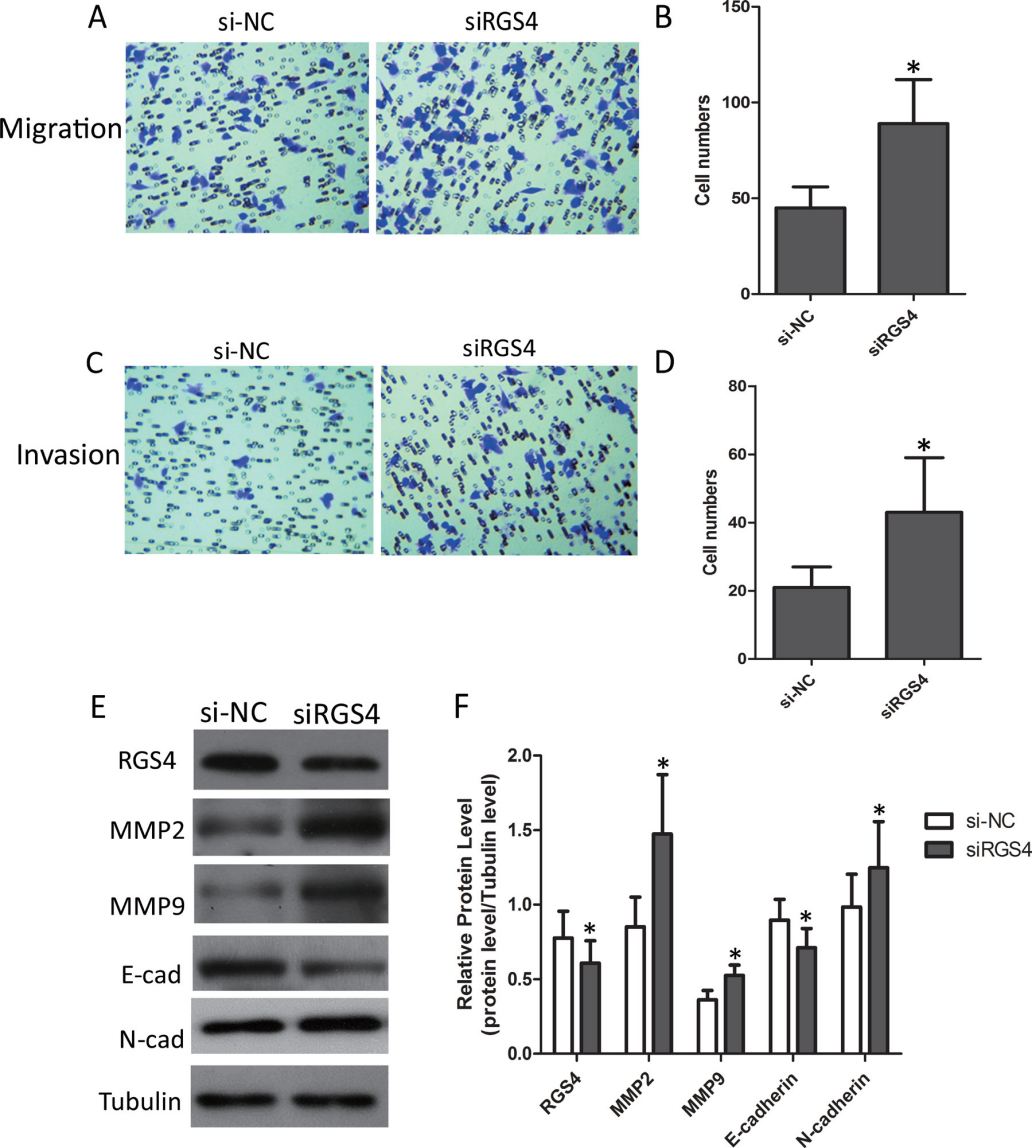
Low expression of RGS4 increases melanoma cell migration and invasion (**A** to **D**) Migration and invasion assays were performed using A375 cell transfected with siRGS4 and corresponding si-NC, respectively. (**E** and **F**) The effect of RGS4 down-regulation on the expression of MMP2,MMP9, E-cadherin and N-cadherin was examined by Western blot. Error bars indicate ± SD. *p < 0.05; **p < 0.01 by Student’s *t*-test. All the results were repeated thrice.

Trans-well assays ± Matrigel demonstrated that A375 cell transfected with siRGS4 had higher invasive activity than control cells (Figure 5A–5D). As in the case above, we also detected the expression of matrix metalloproteinases (MMPs). Western blot results showed that the protein levels of MMP2 and MMP9 were notably up-regulated with low-expression of RGS4. In addition, we further detected the effect of RGS4 on the expression of EMT markers E-cadherin and N-cadherin. Down-regulation of E-cadherin and up-regulation of N-cadherin were observed in RGS4 knockdown A375 cell. Furthermore, downregulation of RGS4 facilitates melanoma cell migration and invasion (Figure 5E and 5F).

### RGS4 plays roles in melanoma cell apoptosis

**Figure 6:**
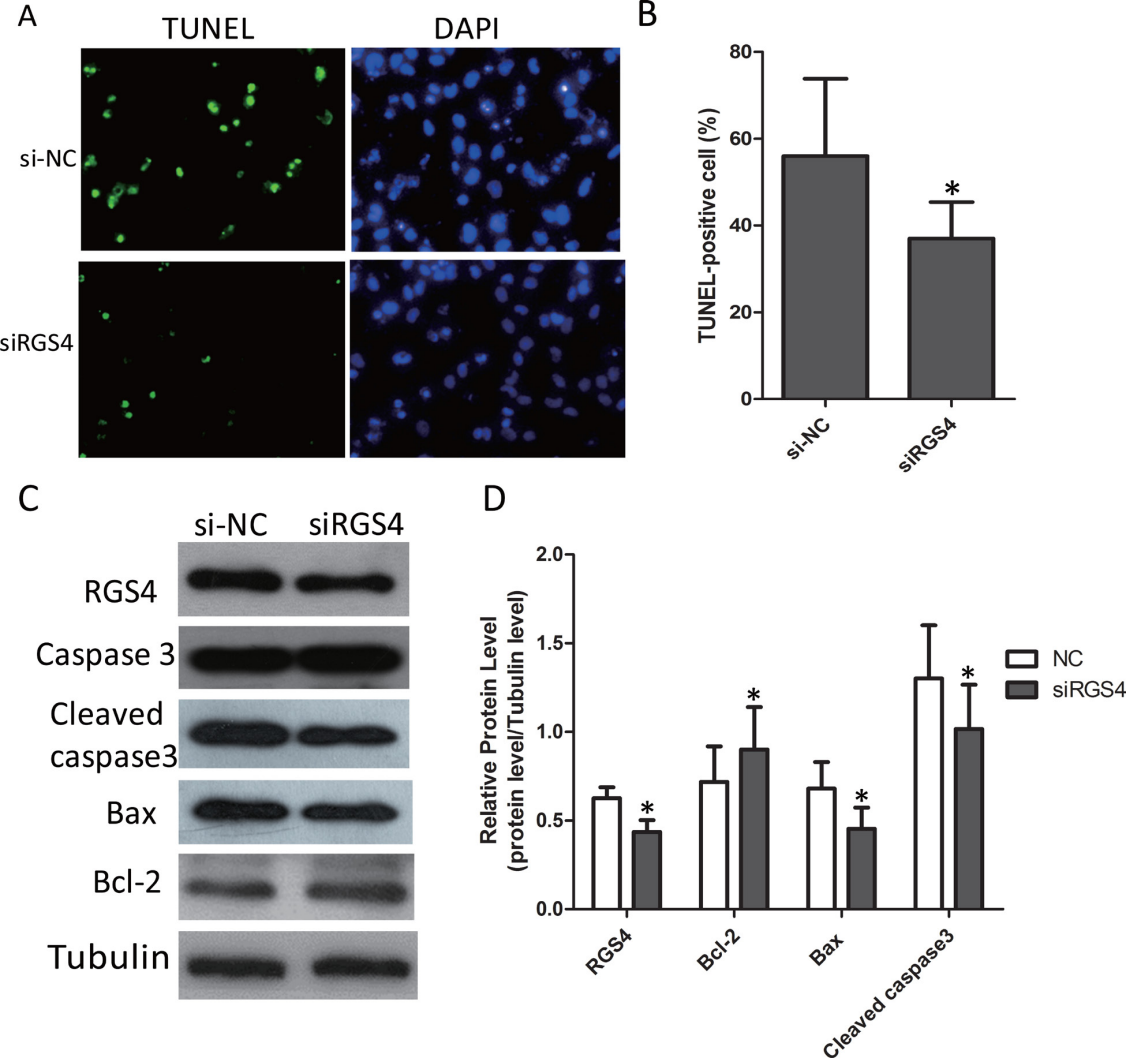
Knockdown of RGS4 significantly suppresses cell apoptosis in melanoma cell lines (**A** and **B**) Comparison of apoptosis ratio of A375 cell transfected with siRGS4 and si-NC by TUNEL assay. (**C** and **D**) The expressions of apoptotic-related proteins, Bcl-2, Bax, total Caspase 3 and Cleaved caspase 3, were detected in A375 cell by Western blot. Error bars indicate ± SD. *p < 0.05; **p < 0.01 by Student’s *t*-test. All the results were repeated thrice.

The effect of RGS4 on apoptosis was investigated by TUNEL assays. After being knocked down by siRGS4, the apoptosis rate was significantly decreased compared with control cells (Figure 6A and 6B). To further confirm this, we detected the expression of apoptotic-related proteins. Western blot results showed that, the expression of anti-apoptotic protein Bcl-2 was increased while the apoptotic protein Bax decreased in siRGS4-transfected cells. In addition, due to the transfection of siRGS4, the expression of Caspase 3 in the apoptotic gene family was reduced (Figure 6C and 6D).

### RGS4 inhibits E2F1 and Cyclin D1 via PI3K/Akt pathway

**Figure 7:**
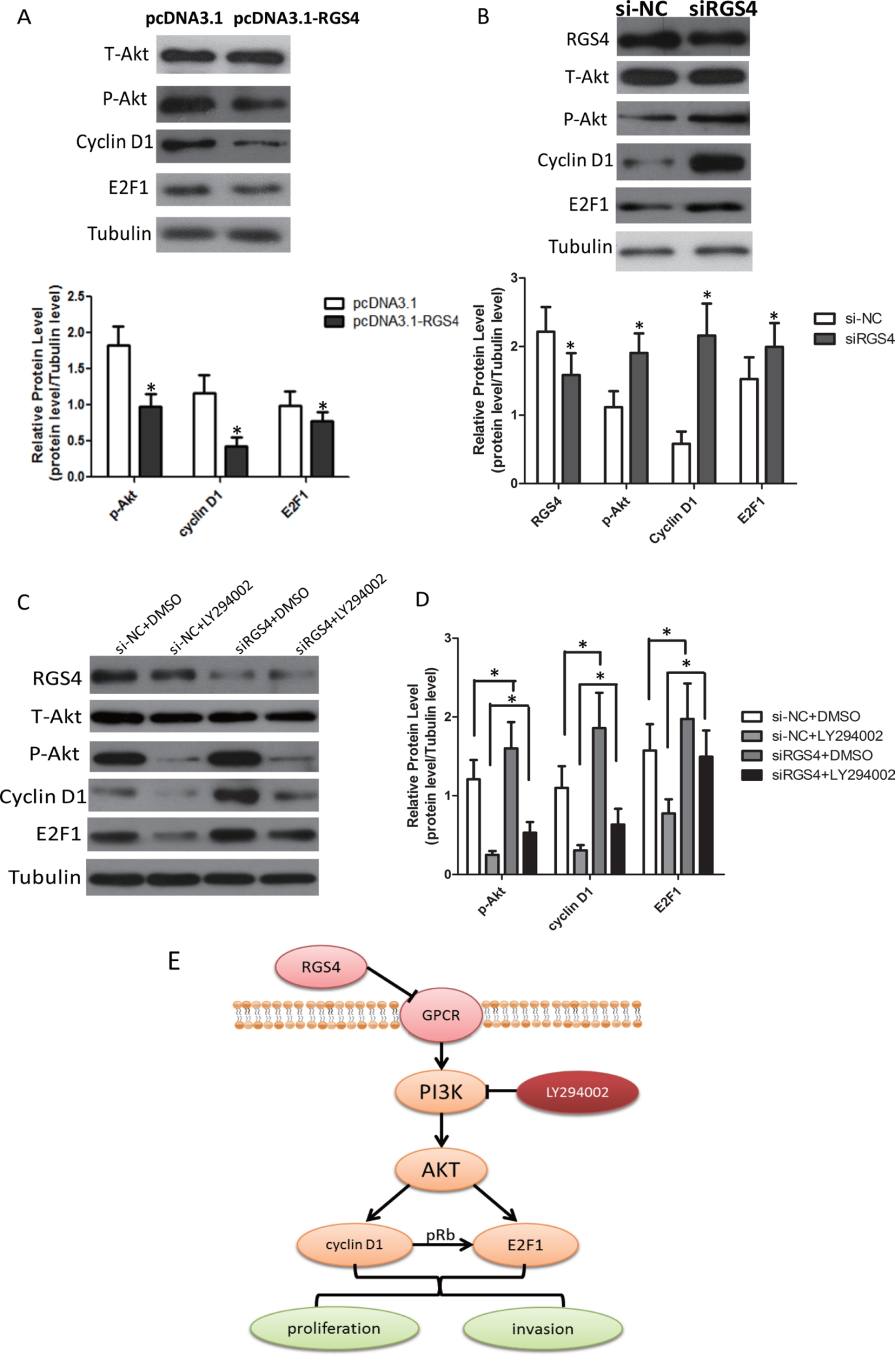
RGS4 regulates E2F1 and Cyclin D1 via PI3K/Akt pathway (**A**) Western blot results show expression changes of T-Akt, p-Akt, Cyclin D1 and E2F1 transfected with pcDNA3.1-RGS4 vector and pcDNA3.1 vector in A375 cell. (**B**) Western blot results show expression changes of RGS4, T-Akt, p-Akt, Cyclin D1 and E2F1 transfected with siRGS4 and si-NC in A375 cell. (**C** and **D**) Western blot results show expression changes of RGS4, T-Akt, p-Akt, Cyclin D1 and E2F1 in siRGS4/A375 and si-NC/A375 after treated with PI3K inhibitor LY294002 (30 μM). (**E**) Model of PI3K/AKT/CyclinD1/E2F1 signaling pathway in melanoma cells. Error bars indicate ± SD. *p < 0.05; **p < 0.01 by Student’s *t*-test. All the results were repeated thrice.

In melanoma cells, several signaling pathways are constitutively activated. Among them, the PI3K/AKT(AKT) signaling pathway is activated by multiple mechanisms and plays a major role in melanoma development and progression [30]. To clarify the signaling pathway in melanoma cells, the effect of RGS4 on p-Akt expression was detected. As expected, the expression of p-Akt was decreased in stable pcDNA3.1-RGS4 transfected A375 cell line (Figure 7A). Meanwhile, RGS4 knockdown markedly stimulated the expression of p-Akt compared to the nagitive control in A375 cell line (Figure 7B). Recent studys showed that PI3K/Akt-dependent Cyclin D1 activation played an essential role in HDL-induced EPC proliferation, migration and angiogenesis [31]. E2F1 and Cyclin D1 are highly expressed in melanoma cells and interrelated to each other [31]. Overexpression of E2F1 has been detected in mantle cell lymphoma and multiple myeloma, which closely related to the up-regulation of Cyclin D1 [32]. Based on these results, it is valuable to analyze the expression of the RGS4 downstream molecules, CyclinD1 and E2F1 under high and low level RGS4 circumstance. Our data indicated that the expression Cyclin D1 and E2F1 was decreased in stable pcDNA3.1-RGS4 transfected A375 cells (Figure 7A) and increased in stable siRGS4 transfected A375 cells. To further validate the signaling pathway, we used PI3K inhibitors LY294002 to interfere with the cells. As shown in Figure 7C and 7D, LY294002 treatment further inhibited the expression of p-Akt, Cyclin D1 and E2F1, while knockdown of RGS4 markedly enhance the effect of LY294002 treatment. These results confirmed that RGS4 inhibited the expression of E2F1 and Cyclin D1 through inactivation of PI3K/Akt pathway, and as a result to inhibit cell proliferation, migration (Figure 7E).

### Knockdown of RGS4 significantly promotes cell proliferation, migration and invasion in M14 cell lines

**Figure 8:**
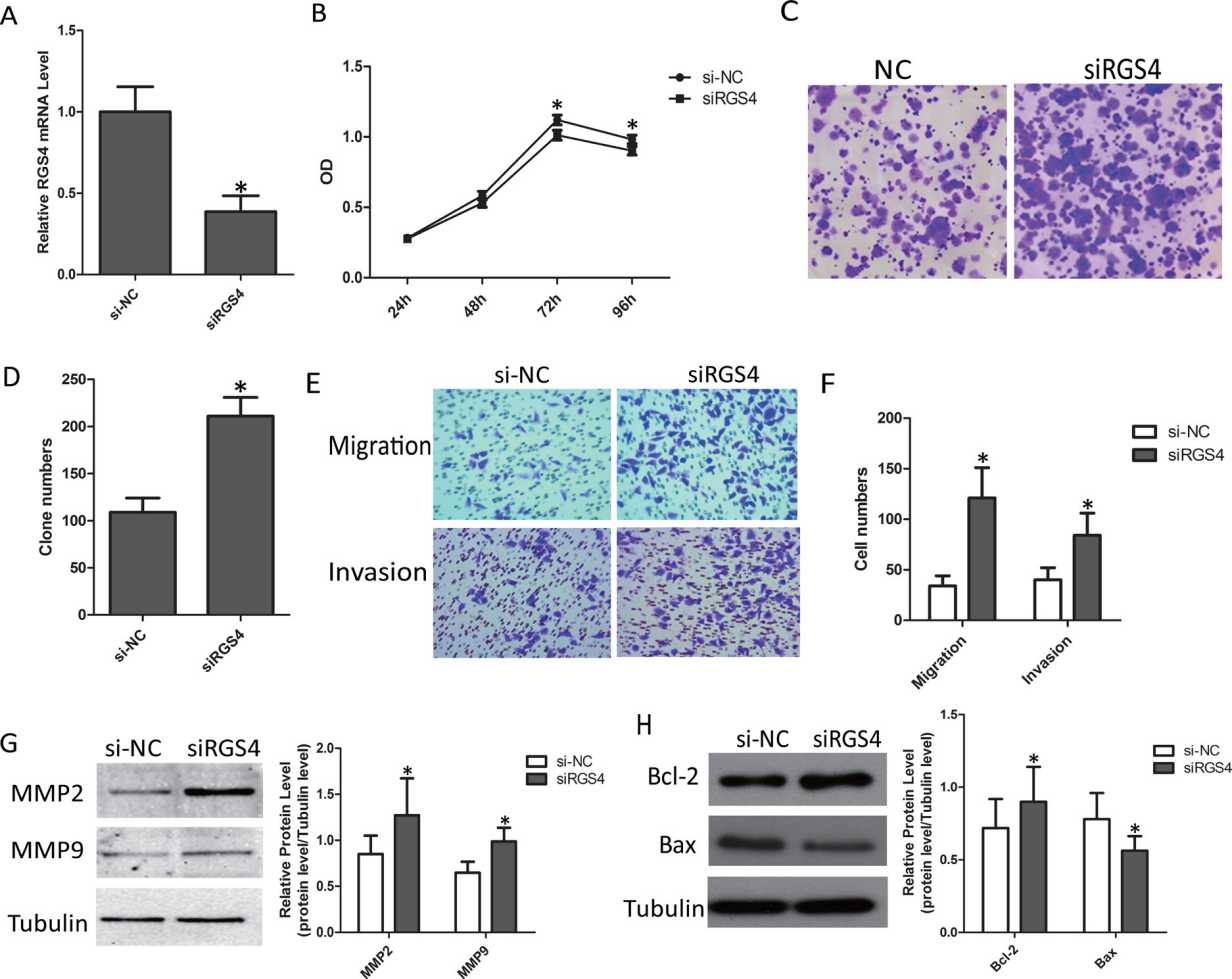
Knockdown of RGS4 significantly promotes cell proliferation, migration and invasion in M14 cell lines (**A**) RGS4 expression in M14 cell transfected with siRGS4 and si-NC assessed qRT-PCR. (**B**) Cell viability of M14 cell was measured by CCK8 assay. (**C**) Colony-forming cells evaluated by colony formation assay. (**D**) Bar charts of positive colonies. (**E** and **F**) Migration and invasion assays were performed using M14 cell transfected with siRGS4 and corresponding si-NC, respectively. (**G**) The effect of RGS4 down-regulation on the expression of MMP2 and MMP9 was examined by Western blot. (**H**) The expressions of apoptotic-related proteins, Bcl-2, Bax, were detected in M14 cell using western blot. Error bars indicate ± SD. *p <0 .05; **p < 0.01 by Student’s *t*-test. All the results were repeated thrice.

To further confirm the effect of knockdown of RGS4 on melanoma, we selected M14 cell line for cell function assays. First, we validated the efficiency of siRGS4. The expression of RGS4 was much lower in siRGS4 transfected M14 cell line (Figure 8A). CCK8 and colony formation assays results showed, M14 cell viability was significantly promoted by downregulation of RGS4 (Figure 8B–8D). Trans-well assays ± Matrigel demonstrated that M14 cell transfected with siRGS4 had higher invasive activity (Figure 8E and 8F). The absent of RGS4 notably up-regulated the protein levels of MMP2 and MMP9 (Figure 8G), suggested that knockdown of RGS4 facilitated M14 cell migration and invasion. Meanwhile, the expression of anti-apoptotic protein Bcl-2 increased while the apoptotic protein Bax decreased in siRGS4-transfected cells, further demonstrating that downregulation of RGS4 can promote M14 cell proliferation (Figure 8H). These results indicated that knockdown of RGS4 promoted the proliferation, migration and invasion of M14 cell.

## DISCUSSION

G protein–coupled receptors (GPCRs), also known as heptahelical receptors, seven-transmembrane domain receptors and G protein–linked receptors (GPLR), form a large protein family of receptors. GPCRs family contain the largest and most diverse group of membrane receptors, which exist only in eukaryotes, including yeast, choanoflagellates and animals [33]. GPCRs have the ability to bind to nucleotides GTP and GDP. Their main function is to induce internal signal transduction pathways by binding molecules outside the cell and as the result to cause cellular responses. G protein–coupled receptors are found to involved in many diseases, which make them are good targets for about 40% of all modern medicinal drugs [8].

The regulator of G protein signaling (RGS) proteins can inhibit different GPCRs signaling by acting as GTPase-accelerating proteins at active G protein subunits [34]. Recently, there are more and more studies on the biochemical and molecular determinants of RGS expression and activity in cancers. These studies especially focus on the R4 RGS protein family, which composes of the smallest RGS proteins in size including RGS1–5, 8, 13, 16, 18 and 21. Studies have shown that the aberrant expression of RGS2 is associated with the tumorigenesis of breast cancer, prostate cancer, ovarian cancer, lymphoma and acute myeloid leukemia [35]. Overexpression of RGS3 promotes apoptosis of leukemic HL-60 cells [36]. RGS5 was reported to reduce ovarian cancer cell proliferation and NSCLC cell metastasis [24, 37]. In addition, RGS16 can inhibit the epidermal growth factor (EGF)-mediated proliferation of breast cancer cells [38]; and it is also associated with lymph node metastasis of pancreatic cancer and may be a prognostic marker for pancreatic cancer [39]. Given that RGS proteins of R4 family play important roles in tumorigenesis, to optimize the function or overexpression of RGS proteins in tumor tissues would be attractive strategies for oncotherapy.

RGS4 is an important member of R4 RGS protein family, and has been widely studied in central nervous system and circulatory system [14]. Meanwhile, the involvement of RGS4 in cancer has also got growing studies. Previous studies indicated that RGS4 notably inhibited invasion and migration of breast cancer cells by its GAP-mediated activity [28]. Consistent inhibition was observed in NSCLC cells [29]. However, the physiological function of RGS4 in malignant melanoma is not well known so far.

For the first time, our studies found that the expression of RGS4 in melanoma tissues was lower than that of melanocyte nevus tissues. This result is consistent with the RGS4 expression pattern in breast tumor and NSCLC [28, 29]. RGS4 expression reduction is significantly correlated with positive lymph node metastasis and advanced TNM stage [29]. In our study, RGS4 expression and TNM stage were interrelated in clinic pathological factors by immunohistochemistry. These results suggest that RGS4 may serve as a potential diagnostic biomarker for malignant melanoma.

Functional study indicated that GPR18, the most abundantly overexpressed orphan GPCR in all melanoma metastases, is constitutively active and inhibits apoptosis, suggesting that GPR18 plays an important role in cancer cell survival and may be considered as potential anticancer targets in metastatic melanoma [40]. In addition, R4 RGS proteins, as the suppressors of GPCR signal, appear to be negatively associated with cancer proliferation and progression ,and regulate the pro-apoptotic factors [23, 24, 36–38, 41]. In our study, we found that overexpression of RGS4 inhibited melanoma cell proliferation and promoted apoptosis in melanoma cells. Meanwhile, knockdown of RGS4 can achieve the completely opposite results. On the other hand, functioning as a tumor suppressor, RGS4 inhibited tumor metastasis and invasiveness by regulating EMT-related markers and MMPs [29]. Matrix metalloproteinases (MMPs) are the primary agents responsible for ECM degradation and their abnormal expression link to the progression of many cancers, including melanoma [42, 43]. In this research, we found overexpression of RGS4 restrained melanoma cell migration and invasion; also decreased the expression of MMPs. And knockdown of RGS4 activated melanoma cell migration and invasion; also degradation of RGS4 increased the expression of MMPs. In addition, it has been validated that MMP2 and MMP9 are implicated in the development and metastasis of cutaneous melanoma [44, 45]. Several evidences indicated that CXCR4/SDF1 promoted prostate cancer cell invasion through MMP9 activation [29]. Therefore, RGS4 may affect the expression of MMPs through GPCR signaling pathway.

Our results showed that, on EMT-related markers,overexpression of RGS4 increased the expression of E-cadherin. In contrast, the knockdown of RGS4 lowers the expression of E-cadherin and increased the expression of N-cadherin. EMT (epithelial mesenchymal transition) is a dynamical process by which immotile epithelial cells lost characteristic of intercellular adhesion, acquire mesenchymal features and obtain increased motility and invasiveness. Activation of EMT plays a crucial role in promoting cancer invasion and metastasis in melanoma [46]. Our studies indirectly confirmed that RGS4 inhibited cell migration and invasion in melanoma by depressing the expression of EMT-related markers. The molecular mechanism of RGS4 on EMT, however, is largely unknown and whether RGS4 regulates EMT via GAP activity or other non-GAP mechanismsis not clear. It is worthful to elucidate the mechanism underlying regulation of EMT by RGS4, which attracts our great interest and attention.

The PI3Ks/AKTs are major effectors downstream to cell surface receptors, such as receptor tyrosine kinases(RTKs) and GPCRs [47, 48]. The PI3K/AKT signaling pathway is a central regulator in a variety of biological processes, including metabolism, migration, survival, autophagy and growth [47]. In our study, we found that overexpression of RGS4 decreased the expression of p-AKT, Cyclin D1 and E2F1. And knockdown of RGS4 increased the expression of p-AKT,Cyclin D1 and E2F1.Cyclin D1 plays roles as allosteric regulators of cyclin-dependent kinase 4 and 6 (CDK4 and CDK6) to regulate cell cycle transition from G1 to S phase [49]. Previous studies revealed that Cyclin D1 was one of the downstream components of PI3K/AKT signaling pathway and actived Cyclin D1 played an important role in cell proliferation, migration and angiogenesis [31, 49]. Therefore, we can say that RGS4 inhibits Cyclin D1 via PI3k/AKT signaling pathway for proliferation, migration of melanoma cells. In conjunction with cyclin-dependent kinases, Cyclin D1 phosphorylates the retinoblastoma protein to release E2F1 [50]. In Human Embryo Lung Fibroblasts (HELF), B[a]P promotes cell cycle progression via intracellular signaling pathway involving Cyclin D1/E2F [51]. It was shown that CBX8, however, might facilitate the transcriptional activity of E2F1 by enhancing the phosphorylation of AKT-p27-RB in K562 leukemia cells [52]. Therefore, we hypothesized that RGS4 directly induced inactivation of E2F1 under the regulation of Cyclin D1 or AKT in melanoma. In addition, treatment of LY294002 on the cells blocked activation of PI3K/AKT, Cyclin D1 and E2F1 due to the knockdown of RGS4 in melanoma cells and consequently decreased the expression of p-Akt, Cyclin D1 and E2F1. In this study, our results supported the hypothesis that RGS4 led to downregulation of Cyclin D1 and E2F1 expression via inactivation of the GPCR-mediated PI3K/AKT pathway, and as the results, inhibited melanoma cell growth, proliferation and so on.

Although our results confirmed the mechanism of RGS4 in melanoma development , the precise mechanism underlying the regulation of RGS4 expression was largely unknown. Previous studies revealed that Transformer-2β (Tra2β), an important splicing factor, increased the relative level of RGS4–1 mRNA isoform and the expression of full-length RGS4 protein. Furthermore, we hypothesized that Cyclin D1 and E2F1, as the important transcription factor, might interact with RGS4 and affected the expression of RGS4. This requires us to further study and explore the mechanism.

In conclusion, for the first time we found that RGS4 expression was downregulated in melanoma tissues and cells which inhibited melanoma cell proliferation, migration and invasion due to the inactivation of Cyclin D1 and E2F1 via GPCR-mediated PI3K/AKT pathway. Our findings suggest that RGS4 might be a new therapeutic target for malignant melanoma.

## MATERIALS AND METHODS

### Immunohistochemistry and evaluation of RGS4 staining

The paraffin-embedded tissue microarray (AlenaBio,China) was dewaxed and rehydrated to retrieve the antigen. Primary antibody against RGS4 (1:200, Proteintech Group) was applied to sections and incubated at 4°C overnight. After washing with PBS, HRP-conjugated secondary antibody was added and further incubated for 2 hrs. The diaminobenzidine (DAB; Beyotime) kit was used to visualize antibody binding. After washing, nuclei were stained with hematoxylin and slides were imaged using microscope (OlympusIX710 Camera, Japan). Cases were scored based on the immunostaining intensity (0, no staining; 1, weak staining; 2, moderate staining; 3, strong staining) and percentage of positive cells (0, < 5%; 1, 5%–25%; 2, 25%–50%; 3, 50%–75%; 4, 75%–100%). Final score was the multiplication of the two above scores so that it ranged from 0 to 12. We defined final scores 0–4 as “low expression” and 5–12 as “high expression”.

### Cell cultures and transfection

The human normal epidermal melanocytes cell line HEM, human melanoma cell line M14, A375, UACC257 and UACC62 were purchased from American Type Culture Collection(ATCC). HEM,M14,A375,UACC257 and UACC62 cells are maintained in DMEM supplemented with 10% fetal bovine serum, penicillin (100 units/mL), and streptomycin (100 Ag/mL; Invitrogen Corp.) at 37°C in a humidified atmosphere with 5% CO2. The PI3k inhibitor, LY294002, was purchased from Selleckchem. The pcDNA3.1-RGS4 vector and the positive control (pcDNA3.1) were purchased from Genechem (Shanghai,China). The siRNA-RGS4 (genOFFTM st-h-RGS4–002) and the negative control (NControl-058) were purchased from RIBOBIO (Guangzhou,China). The cells were plated in six-well plates and transfection was conducted at 70–80% confluence. Transfection used Opti-MEM, serum-free RMPI DMEM and Lipofectamine 2000 (Invitrogen, Carlsbad, CA, USA), according to the transfection instructions. The cells were incubated under normal conditions for 36 hrs at 37°C.

### Quantitative real-time RT-PCR

The total RNA was isolated by using the Trizol reagent (Invitrogen,Carlsbad, CA, USA) followed the common protocal. Two micrograms of the total RNA of each sample was reverse transcribed by using the Ultrapure RNA Kit (CWBIO, Beijing, China) according to the manufacturer’s instructions. Quantitative real-time PCR was performed on an FTC-3000 Real-Time PCR System. The SYBR Green Supermix was used for all of the real-time PCR assays. The thermal cycling conditions for quantitative real-time PCR including an initial denaturation step of 95°C for10 s followed by 40 PCR cycles of 95°C for 5 s, 60°C for 30 s, and 72°C for 10 min. The reaction is terminatedby chilling the mixture to 4°C. All samples were assayed in triplicate in each experiment. The relative amount of mRNA was calculated by using the comparative CT method after normalization to the internal control β-actin mRNA levels. The sequence of primer for the target genes were as follows:

F: 5′-CTTTTTACAGGACGCAGGCAT-3′; R: 5′-CAGCAGGAAACCTAGCCGAT-3′ for RGS4; F: 5′-TGACGTGGACATCCGCAAAG-3′; R: 5′-CTGGAAGGTGGACAGCGAGG-3′ for β-actin

### Antibodies and Western-blot

Antibodies listed below including Bcl-2 (1:1000), Bax(1:1000), Caspase 3 (1:1000), MMP2 (1:500), MMP9 (1:500), E-cadherin (1:250), N-cadherin (1:1000), Akt (1:1000), p-AKT(1:1000), Cyclin D1 (1:10000) and E2F1 (1:1000) were purchased from Abcam (Cambridge, MA, USA). Rabbit polyclonal anti-RGS4 antibody (1:500), anti-rabbit IgG secondary antibodies and anti-mouse IgG secondary antibodies were purchased from Proteintech Group (USA).

Two days after transection, cells were washed with Dulbecco’s Phosphate-Buffered Saline (DPBS, 1X). Cells were lysed with RIPA buffer (140 mm NaCl, 10 mm Tris·Cl, 1% Triton X-100, 0.1% sodium deoxycholate, 0.1% SDS, 1 mM PMSF, plus protease inhibitor mixture, pH 8.0). Cell lysates equal to total protein 30 μg from each sample were subjected to SDS-PAGE (10% acrylamide). After SDS-PAGE, the proteins were transferred to PVDF membranes. Membranes were blocked with TBS containing 5% nonfat milk and 0.1% Tween-20 for 1 hr at room temperature. Primary antibodies were incubated for 2 hrs at room temperature, followed by multiple washes in 1X TBS. The appropriate secondary antibodies (1:2000) were incubated in the blocking solution for 1 hrs at room temperature, followed by multiple washes with TBS. Chemiluminescence was detected using a Super Signal West Femto maximum sensitivity substrate kit (Pierce) in accordance with the manufacturer›s protocol. The assay was repeated for at least three times.

### CCK8 assay

The M14 and A375 cells were plated in a 96-well plate at a concentration of 100 cells per well, respectively. The wells were treated as T1 group (si-NC) and T2 group (si-RGS4). For cell proliferation assay, 10μL of CCK8 solution (Solarbio, Beijing, China) was added to each well, and incubated at 37°C for 2 hrs. The absorbance was measured at a wave length of 450 nm by using the microplate reader (Synergy 2 Multi-Mode Microplate Reader; BioTek, Winooski, VT, USA).

### Colony formation assay

The cells were seeded into 6-well plates at a density of 500 cells/well and incubated for 10 days at 37°C with the growth medium being replaced every 3 days. The colonies were fixed with methanol and stained with 0.5% crystal violet (in methanol:water, 1:1). The number of the colonies containing at least 50 cells was then counted. Data are presented as mean ± SD of at least three independent experiments.

### Trans-well migration and invasion assays

Cell invasion assay was conducted by using Trans-well chambers according to the manufacturer’s instructions. Photomicrographs were captured and the number of cells invaded through the membrane was counted. Experiments were performed for three independent times.

### Tunel assay

The terminal deoxynucleotidyl TUNEL method was performed to label the 3′-terminus of the fragmented DNA of the apoptotic cells according to the manufacturer’s protocol (One Step TUNEL Apoptosis Assay Kit, Beyotime, China). The cell slices were fixed with 4% paraformaldehyde in 1x PBS for 1hr and washing with 1xPBS for 5 minutes 3 times. After permeabilized with1x PBS containing 0.1%Trition X-100 for 2min on ice, cell slices were incubated with TUNEL working solution for 1 hr at 37°C in darkness. After washing, nuclei were stained with DAPI (Invitrogen, USA). The Cy3-labeled TUNEL-positive cells were examined with fluorescence microscopy (Olympus IX710 Camera, Japan). The cells with green fluorescence were considered as apoptotic cells. For counting apoptotic cells, we selected five same size areas of each cell slice to calculate and analyze with the Image Pro Plus soft-ware.

### Statistical analyses

The data are expressed as the mean ± SD and were analyzed with SPSS v11.5 (SPSS Inc., Chicago, IL, USA). Pearson Chi-square test was performed to examine the association of RGS4 and the various clinicopathological factors. The statistical analyses were performed using two-tailed Student’s *t*-test (GraphPad Prism 5). Differences with *p* < 0.05 were considered significant.
